# Human and mouse muscle transcriptomic analyses identify insulin receptor mRNA downregulation in hyperinsulinemia‐associated insulin resistance

**DOI:** 10.1096/fj.202100497RR

**Published:** 2021-12-18

**Authors:** Haoning Howard Cen, Bahira Hussein, José Diego Botezelli, Su Wang, Jiashuo Aaron Zhang, Nilou Noursadeghi, Niels Jessen, Brian Rodrigues, James A. Timmons, James D. Johnson

**Affiliations:** ^1^ Department of Cellular and Physiological Sciences Life Science Institute University of British Columbia Vancouver British Columbia Canada; ^2^ Faculty of Pharmaceutical Sciences University of British Columbia Vancouver British Columbia Canada; ^3^ Department of Biomedicine Aarhus University Aarhus Denmark; ^4^ Steno Diabetes Center Aarhus Aarhus University Hospital Aarhus Denmark; ^5^ Augur Precision Medicine LTD Stirling University Innovation Park Stirling Scotland; ^6^ William Harvey Research Institute Queen Mary University of London London UK

**Keywords:** hyperinsulinemia, insulin receptor, insulin resistance, insulin signaling, SIN3A

## Abstract

Hyperinsulinemia is commonly viewed as a compensatory response to insulin resistance, yet studies have demonstrated that chronically elevated insulin may also drive insulin resistance. The molecular mechanisms underpinning this potentially cyclic process remain poorly defined, especially on a transcriptome‐wide level. Transcriptomic meta‐analysis in >450 human samples demonstrated that fasting insulin reliably and negatively correlated with *INSR* mRNA in skeletal muscle. To establish causality and study the direct effects of prolonged exposure to excess insulin in muscle cells, we incubated C2C12 myotubes with elevated insulin for 16 h, followed by 6 h of serum starvation, and established that acute AKT and ERK signaling were attenuated in this model of in vitro hyperinsulinemia. Global RNA‐sequencing of cells both before and after nutrient withdrawal highlighted genes in the insulin receptor (INSR) signaling, FOXO signaling, and glucose metabolism pathways indicative of ‘hyperinsulinemia’ and ‘starvation’ programs. Consistently, we observed that hyperinsulinemia led to a substantial reduction in *Insr* gene expression, and subsequently a reduced surface INSR and total INSR protein, both in vitro and in vivo. Bioinformatic modeling combined with RNAi identified SIN3A as a negative regulator of *Insr* mRNA (and JUND, MAX, and MXI as positive regulators of *Irs2* mRNA). Together, our analysis identifies mechanisms which may explain the cyclic processes underlying hyperinsulinemia‐induced insulin resistance in muscle, a process directly relevant to the etiology and disease progression of type 2 diabetes.

**Abbreviations**
AKT: AKT serine/threonine kinase, also known as protein kinase B
Aldoa: aldolase, fructose‐bisphosphate A
AS: after starvation
BS: before starvation
ERK: extracellular signal‐regulated kinase (ERK1 and 2 are also known as MAPK3 and 1)
Ets1: ETS proto‐oncogene 1, transcription factor
Fgf1r: fibroblast growth factor receptor 1
FOXO: forkhead box O
G6pc3: glucose‐6‐phosphatase catalytic subunit 3
Hk1: hexokinase 1
IGF1R: insulin‐like growth factor 1 receptor
INSR: insulin receptor
IRMOE: insulin receptor muscle over‐expression
IRS: insulin receptor substrate
JUND: JunD proto‐oncogene, AP‐1 transcription factor subunit
KEGG: Kyoto Encyclopedia of Genes and Genomes
MAX: MYC associated factor X
MXI1: MAX interactor 1, dimerization protein
Myc: MYC proto‐oncogene, BHLH transcription factor
Pck2: phosphoenolpyruvate carboxykinase 2, mitochondrial
Pik3c3: phosphatidylinositol 3‐kinase catalytic subunit type 3
Shc2: Src homology 2 domain‐containing transforming protein 1
SIN3A: SIN3 transcription regulator family member A
T2D: type 2 diabetes

## 1 INTRODUCTION

Hyperinsulinemia and insulin resistance are cardinal features of type 2 diabetes (T2D), yet their co‐association makes it challenging to establish their precise physiological and molecular relationships. Insulin resistance has been widely viewed as the primary cause of T2D, and hyperinsulinemia considered to be a purely compensatory response.^1^, ^2^ However, a growing body of evidence suggests the opposite may be true in many cases.^3^, ^4^, ^5^ Hyperinsulinemia can be observed prior to insulin resistance in the context of obesity and on the road to T2D.^6^, ^7^, ^8^ Elevated insulin can also precede increased body mass index (BMI)^9^ and is associated with future T2D in longitudinal studies.^10^, ^11^ We recently used a loss‐of‐function genetic approach to directly demonstrate that hyperinsulinemia contributes causally to age‐dependent insulin resistance in the absence of hyperglycemia.^12^ Reducing hyperinsulinemia in partial insulin gene knockout mice also prevents and/or reverses diet‐induced obesity in adult mice.^12^, ^13^, ^14^ Further, rodents,^15^, ^16^ healthy humans,^17^, ^18^ and people with type 1 diabetes^19^ subjected to prolonged insulin administration have reduced insulin responsiveness independent of hyperglycemia, strongly implying that relative hyperinsulinemia can self‐perpetuate or cause insulin resistance.

The mechanisms by which hyperinsulinemia can drive insulin resistance remain poorly understood, particularly at the transcriptome‐wide level. Insulin signaling regulates the expression of numerous genes^20^ through kinase signaling cascades that culminate in transcription factor activation.^21^ Euglycemic‐hyperinsulinemic clamp studies have identified genes regulated during acute (~3 h) insulin infusion in vivo.^22^ However, the multiple interacting and time‐dependent effects of the hyperinsulinemic clamp on systematic metabolism make it challenging to identify the direct and lasting effects of elevated insulin in vivo. Cell culture provides a more constrained model for isolating the primary effects of hyperinsulinemia. An early study by Di Camillo et al.^23^ of the time‐dependent transcriptomic responses in muscle cells to physiological insulin (20 nM) identified strong feedback to gene expression in the canonical insulin signaling pathways, yet no impact on the expression of the insulin receptor was reported. It remains unclear how hyperinsulinemia‐induced insulin resistance in a cell model impacts the transcriptome, and whether such changes mimic in vivo observations.

In the present study, we characterized a muscle cell model of hyperinsulinemia‐induced insulin resistance and establish that the transcriptomic changes in our in vitro model are consistent with those observed in human skeletal muscle across a range of insulin resistant states. We further identify transcriptional regulators that play important roles in mediating the effects of hyperinsulinemia, illuminating how hyperinsulinemia contributes to insulin resistance.

## 2 MATERIALS AND METHODS

### 2.1 Cell culture

**FIGURE 1:**
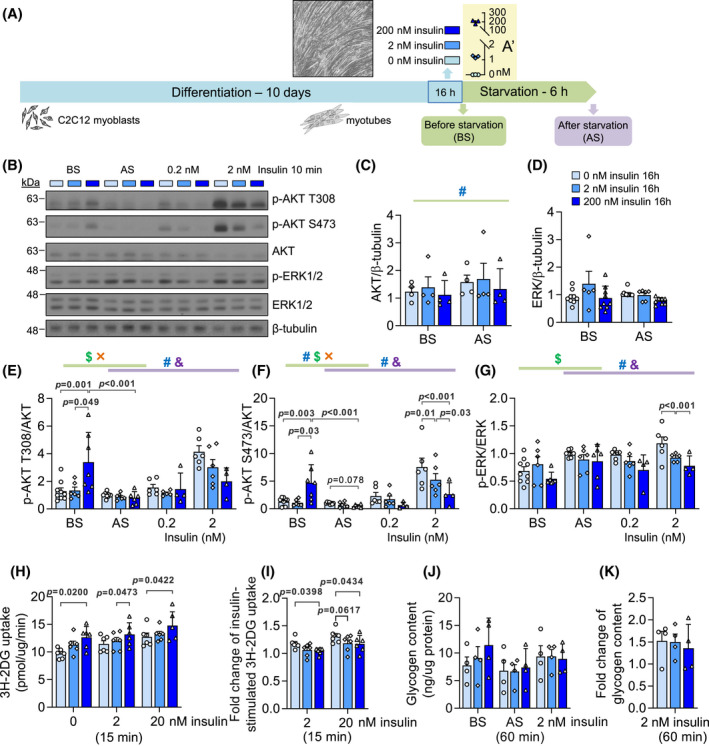
Basal and acute insulin signaling in an in vitro hyperinsulinemia‐induced insulin resistance model. (A) The workflow of C2C12 myotube differentiation, high insulin treatment, and serum starvation. Differentiated myotubes were cultured in control (0 nM insulin) or hyperinsulinemic (2 or 200 nM insulin) medium for 16 h and were analyzed before and after serum starvation. (A') The insulin concentration in the medium at the end of 16‐h high insulin treatment (*n* = 3). (B) Representative western blot images of phospho‐AKT (T308, S473), total AKT, phospho‐ERK1/2, and total ERK1/2. (C) Total AKT and (D) total ERK abundance under high insulin treatments before starvation (BS) and after starvation (AS). (E) Phospho‐AKT (T308), (F) phospho‐AKT (S473) and (G) phospho‐ERK1/2 measurements before starvation (BS), after starvation (AS), and stimulated by 0.2 or 2 nM insulin for 10 min after serum starvation. (*n* = 6–9; ^#^effect of hyperinsulinemia, ^$^effect of starvation, ^&^effect of acute insulin, ^×^interaction between two factors, Mixed Effect Model.) (H) The uptake of 2‐deoxy‐D‐glucose (2DG) under 0, 2, or 20 nM acute insulin for 15 min after serum starvation. (I) The insulin‐stimulated uptake of 2DG in response to 2 or 20 nM acute insulin for 15 min. (*n* = 6) (J) Glycogen content before starvation (BS), after starvation (AS), or after 60‐min 2 nM acute insulin treatment after starvation. (K) The fold change of glycogen content induced by 2 nM acute insulin compared to AS. (*n* = 4)

The C2C12 mouse myoblast (ATCC cell line provided by Dr. Brian Rodrigues, University of British Columbia, Vancouver, Canada) was maintained in Dulbecco's modified Eagle's medium (DMEM, Cat. #11995073, Gibco) supplemented with 10% (v/v) fetal bovine serum (FBS, Gibco), and 1% (v/v) penicillin‐streptomycin (100 μg/ml; Gibco). For downstream analysis, 8 × 10^5^ cells/well of cells were seeded in 6‐well plates and cultured at 37°C under 5% CO_2_. Confluent (90%) myoblasts were differentiated into myotubes by culturing the cells in differentiation medium (DMEM supplemented with 2% horse serum and 1% penicillin‐streptomycin) for 10 days. To induce insulin resistance by hyperinsulinemia in vitro, C2C12 myotubes were cultured in the differentiation medium containing 2 or 200 nM human insulin (Cat.# I9278, Sigma) for 16 h prior to reaching day 10 (Figure 1A). Insulin concentrations after the 16 h hyperinsulinemia treatment were determined using human insulin RIA kit (Millipore). To mimic starvation, myotubes were maintained in the serum‐free medium (DMEM supplemented with 1% penicillin‐streptomycin) for 6 h prior to harvesting. All experiments were repeated with biological replicates using cells from different passages.

### 2.2 Experimental animals

Animal protocols were approved by the University of British Columbia Animal Care Committee in accordance with national guidelines. Isoform specific insulin deficient mice (*Ins1* ^+/+^; *Ins2* ^−/−^ and *Ins1* ^+/−^; *Ins2* ^−/−^) were randomly assigned to be fed *ad libitum* either a high‐fat diet (Research Diets D12492, 20% protein, 60% fat, 20% carbohydrate content, energy density 5.21 Kcal/g, Brunswick, NJ, US) or low‐fat diet (Research Diets D12450B, 20% protein 10% fat, 70% carbohydrate content, energy density 3.82 Kcal/g, Brunswick, NJ, US) for 4 weeks starting from 8 weeks old. Then, at 12 weeks of age, blood fasting glucose was measured using OneTouch Ultra2 glucose meters (LifeScan Canada Ltd, BC, Canada), and serum fasting insulin was assessed using mouse insulin ELISA kit (Alpco Diagnostics, Salem, NH, USA; detection limit 0.0324–1.19 nM), following 4‐h fasting. Lysates of gastrocnemius muscle from mice at 12 weeks old were used in this study.

### 2.3 Western blot analyses

C2C12 myotubes or mice skeletal muscle (gastrocnemius) tissues were sonicated in RIPA buffer (50 mM β‐glycerol phosphate, 10 mM HEPES, 1% Triton X‐100, 70 mM NaCl, 2 mM EGTA, 1 mM Na_3_VO_4_, and 1 mM NaF) supplemented with complete mini protease inhibitor cocktail (Roche, Laval, QC), and lysates were resolved by SDS‐PAGE. Proteins were then transferred to PVDF membranes (BioRad, CA) and probed with antibodies against p‐ERK1/2 (Thr202/Tyr204) (1:1000, Cat. #4370), ERK1/2 (1:1000, Cat. #4695), p‐AKT (Ser473) (1:1000, Cat. #9271), p‐AKT (Thr308) (1:1000, Cat. #9275), AKT (1:1000, Cat. #9272), INSR‐β subunit (1:1000, Cat. #3020S), p‐INSRβ (Tyr1150/1151) (1:1000, Cat. #3024), FOXO1 (1:1000, Cat. #2880), p‐FOXO1 (Thr24) (1:1000, Cat. #9464), all from Cell Signalling (CST), and β‐tubulin (1:2000, Cat. #T0198, Sigma). The signals were detected by secondary HRP‐conjugated antibodies (Anti‐mouse, Cat. #7076; Anti‐rabbit, Cat. #7074; CST) and Pierce ECL Western Blotting Substrate (Thermo Fisher Scientific). Protein band intensities were quantified with Image Studio Lite software (LI‐COR).

### 2.4 Glucose uptake and glycogen content assay

C2C12 myotubes were differentiated, treated with 0, 2, or 200 nM insulin for 16 h, and serum‐starved for 6 h as described above. Then, they were incubated in 0, 2, or 20 nM insulin in serum‐free media for 1 h. Myotubes were washed with Krebs‐Ringer phosphate‐ HEPES buffer (KRPH buffer; 5 mM Na_2_HPO_4_, 10 mM HEPES, 1 mM MgSO_4_, 1 mM CaCl_2_, 136 mM NaCl, 4.7 mM KCl, and 0.2% BSA, pH 7.4). Glucose uptake was then determined by incubating the cells in KRPH buffer containing 0.1 mM 2‐deoxyglucose and 1 µCi/ml of 2‐deoxy‐[3H]glucose for 15 min (37°C, 5% CO_2_). In preliminary tests, 20 µM cytochalasin B, a high affinity inhibitor of glucose transport, was added in some wells to determine nonspecific passive glucose uptake, which was minimal; therefore, this cytochalasin B control was omitted in our experiments. The plate was placed on ice, and the reaction was stopped by washing three times with ice‐cold PBS. Cells were lysed with 0.5M NaOH, neutralized with 0.5M HCl, and an aliquot was taken for liquid scintillation counting to measure labeled glucose uptake (normalized to protein content and expressed as pmol/mg/min). Myotube response to insulin is expressed as the fold change of insulin‐stimulated (2 or 20 nM insulin) to basal active (0 nM insulin) transport rates.

Cellular total glycogen content was determined using a commercially available kit (Abcam, ab65620, colorimetric) according to the manufacturer’s instructions. Briefly, plated C2C12 cells were washed twice with cold PBS, then scraped and homogenized in sterile mqH2O and boiled for 10 min. Samples were centrifuged at 18,000 *g* for 10 min at 4°C, and the supernatant was collected and transferred to new tubes. 50 μl of supernatant was added to a reaction well in a 96 well plate. Glycogen standard (0.2 mg/ml) was used to prepare a standard curve (0 to 2 μg/well). The reaction mix was prepared as described in the assay protocol and after the final incubation with the enzyme mix, a Pherastar FS microplate reader was used to measure absorbance at 570 nm. Data were normalized to protein content.

### 2.5 Linear regressions and dominance analysis

The mice described above included 9 males and 11 females with low fat diet (LFD), and 12 males and 12 females with high fat diet (HFD). The mice were separated into four groups by sex and diet status (male LFD, male HFD, female LFD, female HFD). Skeletal muscle lysates of these four groups were analyzed using four separate SDS‐PAGE to measure INSR (quantified as INSR/tubulin ratios); therefore, we avoided the comparison between these groups but analyzed data within each group. As shown in the gel images (Figure 6A), samples with extremely low protein levels and/or nearly invisible tubulin bands were excluded to avoid skewing the quantification of INSR. Amongst all subjects, two males and four females with LFD and one male and two females with HFD were excluded from analysis due to missing INSR or fasting insulin measurements. Linear regressions were calculated between INSR and one of fasting insulin, fasting glucose or body weight. The multiple linear regression of each group was then calculated, with INSR being the response variable and fasting insulin, fasting glucose and body weight being explanatory variables collectively. These statistical analyses were performed with lm() function in R. Dominance analysis was performed on each multiple linear regression to determine the contribution of each explanatory variable.^24^ Standard errors of means for dominance analysis were calculated and plotted using 10 000 bootstrap resamples. Both steps were performed following the R package ‘dominanceanalysis’.

### 2.6 Surface protein biotinylation assay

Biotinylation of surface proteins was performed as previously described^25^ with modifications (Figure 4A). In brief, cells were incubated with cell‐impermeable EZ‐Link‐NHS‐SS‐biotin (300 μg/ml in PBS; Pierce) at 37°C for 2 min. Cells were then immediately placed on ice and washed with ice‐cold 50 mM Tris‐buffered saline (TBS) to remove excess biotin. For isolating surface proteins, cells were washed using ice‐cold PBS and lysed in complete RIPA buffer (supplemented with complete mini protease inhibitor cocktail (Roche, Laval, QC) and Na_3_VO_4_). For detecting internalized proteins, cells were washed with PBS and incubated in the serum‐free medium supplemented with 0.2, 2, or 20 nM insulin at 37°C to stimulate INSR internalization. After certain time periods, cells were placed on ice, washed with ice‐cold PBS, incubated with Glutathione solution (50 mM glutathione, 75 mM NaCl, 1 mM EDTA, 1% BSA, 75 mM NaOH) for 20 min to strip the remaining surface biotin, washed with excess PBS, and lysed in complete RIPA buffer. Lysates were quantitated and incubated with NeutrAvidin beads (Pierce) overnight at 4°C to isolate biotinylated surface or internalized proteins. Biotinylated proteins were eluted from the NeutrAvidin beads by boiling in Blue Loading Buffer (CST) containing 50 mM DTT for 5 min. Surface or internalized INSR in eluent and total INSR in lysates were detected in western blot analysis.

### 2.7 siRNA knockdown in C2C12 myoblasts

All siRNAs are from Thermo Fisher Scientific with the specific Assay IDs as follows: *Foxo1* (MSS226201), *Sin3a* (151684), *Elf1* (157302), *Mxi1* (68202), *Myc* (68302), *Ets1* (101877), *Hcfc1* (158001), *Nrf1* (68266), *Jund* (67635), *Ctcf* (60925), *Max* (155266), *Maz* (501159), and Silencer Cy3‐labeled Negative Control No.1 siRNA (AM4621). siRNAs were transfected into C2C12 myoblasts using the Lipofectamine RNAiMAX reagent (Invitrogen) according to the manufacturer's instructions with 50 pmol of siRNA and 4 µl of transfection reagent per well of a 12‐well plate.

### 2.8 RNA isolation and quantitative real‐time PCR analysis

Total RNA was isolated from both control and high insulin‐treated C2C12 myotubes before and after serum starvation or C2C12 myoblasts post siRNA transfection using the RNEasy mini kit (Qiagen). cDNA was generated by reverse transcription using qScript cDNA synthesis kit (Quanta Biosciences, Gaithersburg, MD, USA). Transcript levels of target genes in the equal amount of total cDNA were quantified with SYBR green chemistry (Quanta Biosciences) on a StepOnePlus Real‐time PCR System (Applied Biosystems). All data were normalized to *Hprt* by the 2^−ΔCt^ method. The following primers are used in qPCR: *Insr*‐*A*/*B* forward 5′‐TCC TGA AGG AGC TGG AGG AGT‐3′, *Insr*‐*A* reverse 5′‐CTT TCG GGA TGG CCT GG‐3′, *Insr*‐*B* reverse 5′‐TTC GGG ATG GCC TAC TGT C‐3′^26^; *Insr* (in siRNA experiments) forward 5′‐TTT GTC ATG GAT GGA GGC TA‐3′ and reverse 5′‐CCT CAT CTT GGG GTT GAA CT‐3′^27^; *Igf1r* forward 5′‐GGC ACA ACT ACT GCT CCA AAG AC‐3′ and reverse 5′‐CTT TAT CAC CAC CAC ACA CTT CTG‐3′^26^; *Hprt* forward 5′‐TCA GTC AAC GGG GGA CAT AAA‐3′ and reverse 5′‐GGG GCT GTA CTG CTT AAC CAG‐3′^28^; *Foxo1* forward 5′‐CCC AGG CCG GAG TTT AAC C‐3′ and reverse 5′‐GTT GCT CAT AAA GTC GGT GCT‐3′, *Tbp* forward 5′‐AGA ACA ATC CAG ACT AGC AGC A‐3′ and reverse 5′‐GGG AAC TTC ACA TCA CAG CTC‐3′, *Nrf1* forward 5′‐TAT GGC GGA AGT AAT GAA AGA CG‐3′ and reverse 5′‐CAA CGT AAG CTC TGC CTT G TT‐3′, *Jund* forward 5′‐GAA ACG CCC TTC TAT GGC GA‐3′ and reverse 5′‐CAG CGC GTC TTT CTT C AGC‐3′, *Ctcf* forward 5′‐GAT CCT ACC CTT CTC CAG ATG AA‐3′ and reverse 5′‐GTA CCG TCA CAG GAA CAG GT‐3′, *Mxi1* forward 5′‐AAC ATG GCT ACG CCT CAT CG‐3′ and reverse 5′‐CGG TTC TTT TCC AAC TCA TTG TG‐3′, *Elf1* forward 5′‐TGT CCA ACA GAA CGA CCT AGT‐3′ and reverse 5′‐CAC ACA AGC TAG ACC AGC ATA A‐3′, *Ets1* forward 5′‐TCC TAT CAG CTC GGA AGA ACT C‐3′ and reverse 5′‐TCT TGC TTG ATG GCA AAG TAG TC‐3′, *Maz* forward 5′‐GCC CCA GTT GCA TCT GTC TT‐3′ and reverse 5′‐CTT CGG AGG TTG TAG CCG TT‐3′, *Max* forward 5′‐ACC ATA ATG CAC TGG AAC GAA A‐3′ and reverse 5′‐GTC CCG CAA ACT GTG AAA GC‐3′, *Myc* forward 5′‐ATG CCC CTC AAC GTG AAC TTC‐3′ and reverse 5′‐CGC AAC ATA GGA TGG AGA GCA‐3′, *Hcfc1* forward 5′‐CGG CAA CGA GGG GAT AGT G‐3′ and reverse 5′‐TAG GCG AGT ACC ATC ACA CAC‐3′, *Sin3a* forward 5′‐GCC TGT GGA GTT TAA TCA TGC C‐3′ and reverse 5′‐CCT CTT GCT CAG TCA AAG CTG‐3′, *Irs2* forward 5′‐CTG CGT CCT CTC CCA AAG TG‐3′ and reverse 5′‐GGG GTC ATG GGC ATG TAG C‐3′ (PrimerBank, https://pga.mgh.harvard.edu/primerbank/).

### 2.9 RNA sequencing and bioinformatic analysis

Total RNA isolated from both control and 200 nM insulin‐treated C2C12 myotubes before and after serum starvation (4 groups, *n* = 5 each group) were sequenced by BRC Sequencing Core at the University of British Columbia. Sample quality control was performed using the Agilent 2100 Bioanalyzer. Qualifying samples were then prepped following the standard protocol for the NEBNext Ultra II Stranded mRNA (New England Biolabs). Sequencing was performed on the Illumina NextSeq 500 with Paired‐End 42 bp × 42 bp reads. Sequencing data were demultiplexed using Illumina's bcl2fastq2. De‐multiplexed read sequences were then aligned to the Mus Musculus mm10 reference sequence using STAR aligner.^29^ Assembly was estimated using Cufflinks (http://cole‐trapnell‐lab.github.io/cufflinks/) using methods available on the Illumina Sequence Hub and with default settings.

Raw counts of the gene reads were filtered for minimal expression by only keeping genes with more than 5 raw reads in more than 5 samples. After normalizing counts by variance stabilizing transformation, differential expression analysis was performed using DESeq2 package using the Benjamini & Hochberg adjusted *p*‐value < .05 as the cutoff.^30^ Raw counts of the mouse skeletal muscle data sets (IRMOE^31^ and Clamp^22^) were analyzed using the same criteria. Kyoto Encyclopedia of Genes and Genomes (KEGG) pathway enrichment analyses were performed using the R package clusterProfiler.^32^ Reactome pathway enrichment analyses were performed using the R package ReactomePA.^33^ The background gene list was set to be all the detectable genes passing the minimal expression filter. Redundant pathways, containing many overlapping genes, were omitted in the figures but are included in supplementary tables. Transcription factor (TF)‐gene network was derived from the ENCODE ChIP‐seq data and illustrated by a visual analytic platform NetworkAnalyst 3.0 (http://www.networkanalyst.ca/).^34^ Top 30 TFs with the highest degree of connections (rank order) with differentially expressed genes were selected for siRNA knockdown.

### 2.10 Human muscle transcriptomic meta‐analyses

We carried out a meta‐analysis using three independent human skeletal muscle transcriptomic data sets (*n* = 488) (Table 1). In all cases, insulin values were log transformed prior to any analysis and were normally distributed under these conditions. Pearson correlation coefficient (*R*) between fasting insulin and normalized gene expression was calculated. Genes with significant correlation (adjusted *p* < .05, |*R*| > 0.2) were selected for further comparisons. *p*‐Values were adjusted for multiple comparisons using the Benjamini & Hochberg method.

**TABLE 1:** Phenotypes of human cohorts

Data sets	Insulin (pM)		Glucose (mM)		Age		Sex	
	Mean	SD	Mean	SD	Mean	SD	Female	Male
SMP	68.1	42.7	5.02	0.69	43.3	15.0	102	89
FUSION	59.1	34.9	6.27	0.78	60.0	7.6	115	163
Møller et al.	176.8	150.7	6.99	1.41	60.8	6.6	7	12

We utilized a large tiling‐type microarray data set, referred to as SMP (STRRIDE‐PD and METAPREDICT studies), which consisted of 191 sedentary individuals with increased BMI and/or impaired glucose tolerance. Gene expression profiles (HTA 2.0 array) and plasma insulin values (K6219 Dako high‐sensitivity enzyme‐linked immunosorbent assay (ELISA)) were generated in a single core lab.^35^ Gene expression was normalized using IRON^36^ producing log2 transformed data for 53 032 probe‐sets representing the detectable protein coding Ensembl transcripts and the Benjamini & Hochberg method was used to adjust the *p*‐values obtained from linear regression (adjusted for age). For the exon‐level analysis of the insulin receptor, expression values were extracted using an EXON‐level CDF, and univariate linear analysis. Full details of all pre‐processing and statistical methods can be found in Refs. [35, 37].

Two RNA‐seq muscle tissue cohorts were utilized. A large study, FUSION (Finland‐United States Investigation of NIDDM Genetics Study; dbGaP accession phs001048.v2.p1), consisted of individuals (*N* = 299) classified into normal glucose tolerance (NGT), impaired fasting glucose (IFG), impaired glucose tolerance (IGT), or type 2 diabetes (T2D). In total, 278 RNAseq samples (HiSeq2000 using 101 bp paired‐end reads) passed QC and were correlated with fasting insulin with muscle RNA. Insulin was measured in serum using the Architect chemiluminescent microparticle immunoassay. A second, case‐control human RNA‐seq data set (Møller et al.) is a small cohort (*N* = 19) consists of age‐matched health subjects (Control), T2D subjects with severe insulin resistance under insulin injection (T2D‐SI group), or oral anti‐diabetic drug (T2D‐OAD group).^38^, ^39^ Serum insulin was analyzed using time‐resolved immunofluorometric assay (AutoDELFIA, PerkinElmer, Finland). Raw counts of the RNA‐seq gene reads were normalized by variance stabilizing transformation.

### 2.11 Statistics

Data were presented as mean ± SEM in addition to the individual data points. A significance level of adjusted *p* < .05 was used throughout. All western blot quantifications (protein band intensity) were analyzed using linear regression modeling^40^ in R Studio 3.4.1. Linear mixed effect models (R package – lme4) were fitted using restricted maximum likelihood.^40^, ^41^ Predictor variables were included as fixed effects, and sample IDs were included as random effects. Mixed effect modeling was used to account for repeated sample measurements and missing data.^40^ Where the random effect was not significant, linear fixed effect modeling was used. Heteroscedasticity and normality of residuals were analyzed used Levene's test and the Shapiro–Wilk test, respectively. Predictor variables, insulin treatment (overnight and acute) and time, were treated as ordinal factors and continuous factors, respectively. The outcome variable, protein band intensity, was treated as a continuous factor and log‐transformed when residuals are not homoscedastic and/or normally distributed. Other comparisons between two categorical variables were performed using Two‐way ANOVA followed by Tukey's multiple comparisons tests, which were conducted in GraphPad Prism (version 9.0.2).

## 3 RESULTS

### 3.1 Hyperinsulinemia induces insulin resistance in muscle cells in vitro

Circulating insulin in humans oscillates in a range between approximately 0.01 and 0.75 nM.^35^, ^42^, ^43^, ^44^, ^45^, ^46^ Although there is no standard criteria for hyperinsulinemia, fasting insulin higher than 0.085 nM is considered to be associated with insulin resistance.^43^ As for mice, fed insulin is ~0.2 nM in lean mice and ~3–5 nM in extremely obese mice such as the *Lep* ^ob/ob^ strain.^47^ To establish an in vitro model of hyperinsulinemic conditions (i.e. in vitro hyperinsulinemia), we incubated differentiated mouse C2C12 myotubes for 16 h in a physiologically high insulin dose of 2 nM or supraphysiological high dose of 200 nM (Figure 1A). Hyperinsulinemia was confirmed after treatment with high insulin (Figure 1A'). After 6 h of serum starvation, insulin signaling was assessed by measuring the phosphorylation of AKT and ERK proteins, two major insulin signaling nodes.^48^ As alterations in the basal state of the insulin signal transduction network have also been reported in hyperinsulinemic humans and animals,^49^ we measured the effects of hyperinsulinemia on AKT and ERK phosphorylation before serum starvation (BS) and after serum starvation (AS). These experiments showed that total AKT protein was downregulated by prolonged 200 nM, but not 2 nM, insulin treatment, while ERK abundance was not altered (Figure 1B–D). After prolonged 200 nM insulin exposure—and before serum starvation—AKT phosphorylation at threonine (T) 308 and serine (S) 473 was elevated, while ERK phosphorylation was unaffected (Figure 1E–G). Of note, phosphorylation of ERK1/2 was increased by serum starvation alone, as previously reported in other cell types (Figure 1G).^50^ Acute AKT and ERK signaling in response to 2 nM acute insulin exposure was reduced by hyperinsulinemia treatment in an insulin dose‐dependent manner (Figure 1E–G). We also characterized the insulin dose‐ and time‐dependent signaling in our in vitro hyperinsulinemia model (200 nM insulin). Phosphorylation of AKT and ERK1/2 were reduced under 0.2, 2, and 20 nM insulin conditions (Figure S1A,B). To put our results in the context of a classical insulin action, we accessed the uptake of 2‐deoxy‐D‐glucose and glycogen synthesis in this hyperinsulinemia model (Figure 1H–K). In the 200 nM hyperinsulinemia group, basal (0 nM acute insulin) glucose uptake was higher (Figure 1H), but the fold change of insulin‐stimulated glucose uptake was lower (Figure 1I). The 2 nM hyperinsulinemia group had numerically less insulin‐stimulated glucose uptake (Figure 1H,I). The glycogen synthesis had large variance and was not significantly different between the groups (Figure 1J,K). Overall, the results establish that robust muscle cell insulin resistance is induced by 16 h of hyperinsulinemia in vitro.

### 3.2 Insulin signaling genes are modulated by hyperinsulinemia and serum starvation in muscle cells

**FIGURE 2:**
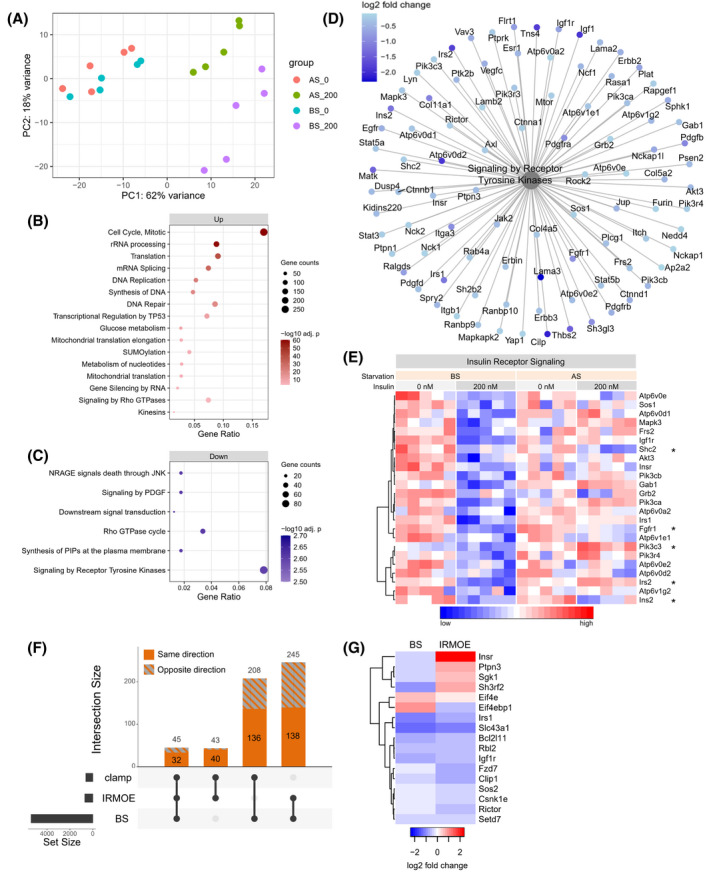
Transcriptomic analysis of hyperinsulinemia and serum starvation. (A) Principal component analysis (PCA) of RNAseq data from 4 groups of treatments, including 0 or 200 nM prolonged insulin both before and after serum starvation. (B,C) Selected top Reactome pathways enriched from (B) upregulated or (C) downregulated genes by hyperinsulinemia before starvation (BS, 0 vs. 200 nM insulin). (D) Differentially expressed genes (BS, 200 vs. 0 nM) enriched under Reactome pathway ‘Signaling by Receptor Tyrosine Kinases’. (E) The normalized gene expressions of downregulated insulin signaling genes before starvation (BS, 200 vs. 0 nM). *Genes that are also differentially expressed after starvation (AS, 200 vs. 0 nM). (F) The number of differentially expressed genes shared between our in vitro study (BS), IRMOE study, and hyperinsulinemic clamp study (clamp). The total number of genes in the intersections are labeled at the top of each bar, and the number of genes altered in the same direction are shown in solid color. (G) The log2 fold change of the genes of interest that altered in both our in vitro model (BS, 200 vs. 0 nM) and IRMOE muscle

To investigate the molecular mechanisms of hyperinsulinemia‐induced insulin resistance, we conducted well powered RNA sequencing (RNA‐seq) on cells exposed to prolonged insulin and serum starvation. We compared the transcriptomes across 4 treatment groups (*n* = 5 per group, 0 or 200 nM insulin, both before and after serum starvation). The transcriptional changes before serum starvation (BS) reveal the effects of chronic insulin, while the transcriptional changes after serum starvation (AS) indicate which transcriptional responses persist or reverse during the 6‐h resting period without insulin. Principal component analysis (PCA) of global gene expression showed that the majority of experimental variation was related to hyperinsulinemia (PCA1), while the impact of starvation was evident in PCA2 (greatly enhanced by preceding hyperinsulinemia conditions) (Figure 2A). There were 2882 up‐regulated and 2506 down‐regulated gene transcripts before starvation (BS, 200 vs. 0 nM) (Table S1), and the top 50 most significantly altered genes were shown in Figure S2A. We categorized the functions of the regulated transcripts using pathway enrichment analyses (Reactome and KEGG, Figures 2B,C and S2B, Tables S2 and S3). The genes upregulated by hyperinsulinemia (BS, 200 vs. 0 nM) related to cell cycle, RNA biology, translation, and glucose metabolism pathways (Figure 2B, Tables S2 and S3), while the downregulated genes were enriched in a wide range of signaling pathways (Figures 2C and S2B, Tables S2 and S3).

Serum starvation after hyperinsulinemia (200 nM, BS vs. AS) changed some of these pathways in the opposite direction, while some pathways remained in the same direction (AS, 200 vs. 0 nM) (Table S2). Serum starvation after hyperinsulinemia resulted in 4356 differentially expressed genes (Table S1); 2704 of those genes overlapped with the differentially expressed genes before starvation (BS, 200 vs. 0 nM), but 2569 of those genes were changed in the opposite direction (Figure S2C). Many upregulated genes related to glucose metabolism were upregulated by hyperinsulinemia, such as *G6pc3*, *Hk1*, *Pck2* and *Aldoa*, some of which were reverted to baseline by starvation (Figure S2D,E). Interestingly, many downregulated genes were linked to the Reactome pathway titled ‘Signaling by Receptor Tyrosine Kinase’ (FDR = 0.002) (Figure 2C,D), and a subset of genes of interest related to insulin receptor signaling were highlighted (Figure 2E). Most of these insulin signaling genes recovered after serum starvation, such as *Insr* and *Irs1*; some remain downregulated, such as *Shc2* and *Fgf1r*; some were upregulated instead, such as *Irs2* and *Pik3c3* (Figure 2E). There were 2481 differentially expressed genes after serum starvation (AS, 200 vs. 0 nM). Despite the recovery effect of serum starvation, 1720 of those genes were also changed before serum starvation (BS, 200 vs. 0 nM), and 1547 of those genes remained to be altered in the same direction (Figure S2F), demonstrating some long‐lasting effects of hyperinsulinemia. Overall, prolonged hyperinsulinemia and insulin removal revealed strong, reciprocal transcriptomic effects. Many insulin signaling genes were reprogramed by hyperinsulinemia, and this may contribute to the insulin resistance in our model.

To support the relevance of our hyperinsulinemia‐induced transcriptomic changes, we compared the RNA responses in our in vitro model with two mouse skeletal muscle systems with sustained insulin signaling. One recent study with insulin receptor muscle over‐expression (IRMOE) demonstrated similarities with our in vitro model, such as increased basal AKT phosphorylation and impaired insulin‐stimulated AKT phosphorylation, indicative of a significant level of post‐receptor insulin resistance.^31^ The second study used the hyperinsulinemic‐euglycemic clamp, in which ~1 nM insulin plasma insulin was maintained for 3 h (high insulin vs. saline),^22^ which represents relatively acute hyperinsulinemia. Compared with IRMOE and clamp studies, our in vitro hyperinsulinemia model had different subsets of overlapping differentially expressed genes (Figures 2F and S3A,B, Table S4). There were 136 differentially expressed genes in our model that were altered in the same direction only in the clamp study but not the IRMOE study (Figure 2F, Table S4), which might represent the functional transcriptomic effects of insulin. There were 138 differentially expressed genes in our model that were altered in the same direction only in the IRMOE study but not the clamp study (Figure 2F, Table S4), which might associate with insulin resistance. While these overlaps are modest, among the common differentially expressed genes between our in vitro model and the IRMOE system, several key genes were consistently altered, such as *Irs1*, *Igf1r*, *Sos*, and *Rictor* (Figure 2G). It appears that our in vitro model represents many features observed with excess insulin signaling in vivo.

### 3.3 Transcriptomic analysis of human skeletal muscle reveals genes associated with fasting insulin including *INSR*

**FIGURE 3:**
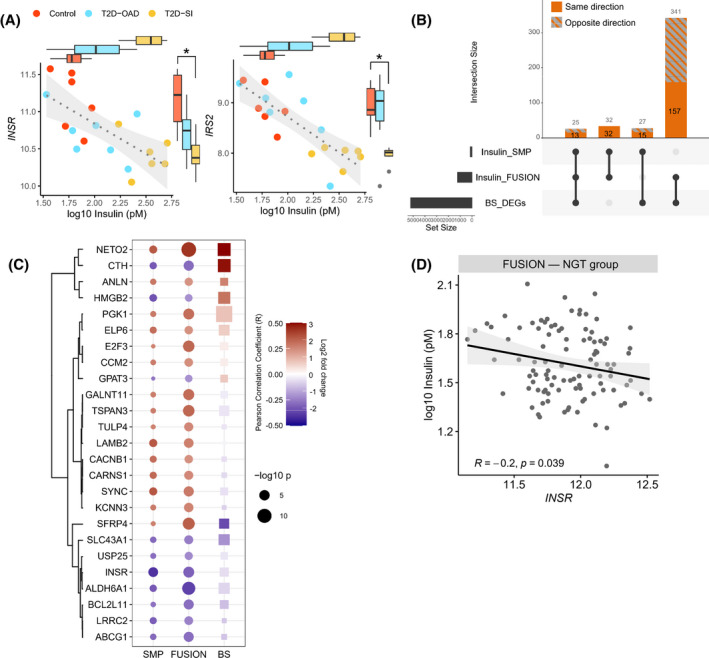
Correlation between fasting insulin and human transcriptome highlights insulin receptor expression. (A) The *INSR* and *IRS2* expressions and the corresponding fasting insulin levels in control subjects, diabetic subjects with oral antidiabetic drug (T2D‐OAD) or with severe insulin resistance (T2D‐SI). (B) The number of genes correlated with fasting insulin or differentially expressed in our in vitro model (BS_DEGs) shared between data sets. The total number of genes in the intersections are labeled at the top of each bar, and the number of genes altered in the same direction are shown in solid color. (C) The common genes in all 3 data sets and their Pearson correlation coefficient (*R*) in human data sets or log2 fold change in our in vitro hyperinsulinemia model (BS). (D) The correlation between *INSR* and insulin in normal glucose tolerance (NGT) group in the FUSION human cohort

We established above that a key feature of excess insulin signaling was the down‐regulation of components of proximal insulin signaling, including the insulin receptor gene, *Insr*. To explore if these specific molecular responses were consistent in humans, we modeled the relationship between fasting insulin and the gene expression in human skeletal muscle (Table 1).^35^, ^38^, ^51^ The human cohorts we studied represent the full range of insulin resistance and cover normal glucose tolerance,^35^, ^38^ pre‐diabetes and diabetes^35^, ^38^ to extreme obesity‐related insulin resistance.^51^ One of the human cohorts had a higher range of fasting insulin (Figure S3C) because the study included diabetic living with diabetes and severe insulin resistance needing insulin injection (T2D‐SI group).^38^ Although correlation analysis is not ideal when applied to small case‐controlled studies, the range of insulin values overlapped and we observed that *INSR* and *IRS2* gene expression levels had a negative correlation with fasting insulin (Figure 3A). Using the two larger human cohorts, after cross‐referencing with orthologous mouse genes in our hyperinsulinemia model, we identified genes that were significantly correlated with fasting insulin and differentially expressed in our hyperinsulinemia model (Figure 3B,C, Table S5). Compellingly, *INSR* was negatively correlated with fasting insulin in all data sets (Figure 3C), consistent with our in vitro model and our previous reports using a HOMA2‐IR model.^35^ *INSR* and insulin also had a negative correlation in the normal glucose tolerance group in the FUSION human cohort (Figure 3D), suggesting that this consistent association is independent of pathological changes or diabetes.

### 3.4 Hyperinsulinemia reduces both *Insr* isoform A and B alongside FOXO1 inhibition

**FIGURE 4:**
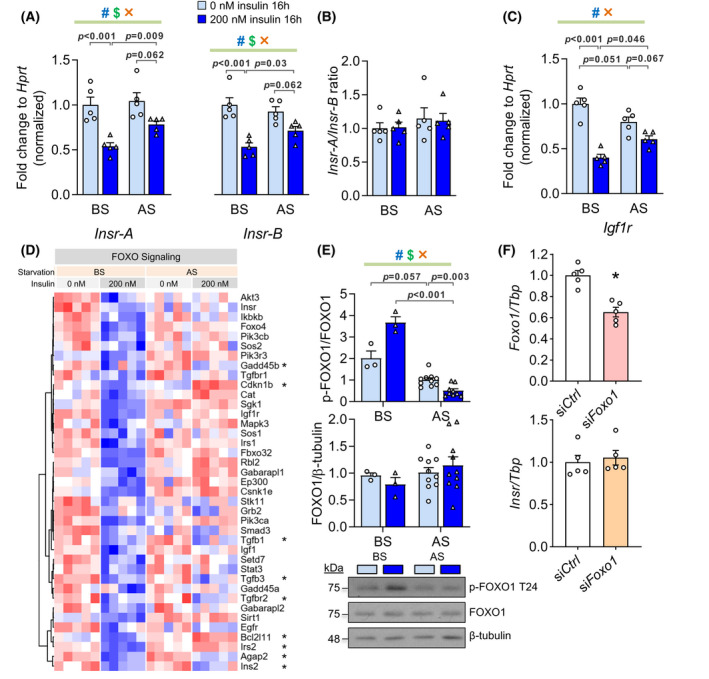
Effects of prolonged hyperinsulinemia and starvation on *Insr* transcription and FOXO1 phosphorylation in vitro. (A) The mRNA levels of *Insr* isoform A or B *(Insr*‐*A or B)* before and after starvation (BS and AS) assessed by qPCR. (B) *Igf1r* mRNA level. (C) The ratio of *Insr*‐*A* to *Insr*‐*B* mRNA (*n* = 5). (D) Normalized counts of downregulated genes under KEGG pathway ‘FOXO signaling’ before starvation (BS, 200 vs. 0 nM). *Genes that are also differentially expressed after starvation (AS, 200 vs. 0 nM). (E) Total and T24 phosphorylation of FOXO1 (*n* = 3 in BS group, *n* = 10 in AS group). (F) mRNA levels of *Foxo1* and *Insr* after knocking down Foxo1 by siRNA (*n* = 5, *p < .05). (^#^effect of hyperinsulinemia, ^$^effect of starvation, ^×^interaction between two factors, 2‐ANOVA)

Loss of *INSR* expression reduces the effects of insulin at a critical component at the very beginning of the insulin signaling cascade. Downregulation of in *Insr* expression as well as alternative splicing in some,^52^, ^53^, ^54^ but not all analysis^35^ has been associated with hyperinsulinemia. Therefore, we carried out a detailed analysis of transcription from the *Insr* gene in our cell culture model, using qPCR. The isoform A and B of *Insr* (*Insr*‐*A* and *Insr*‐*B*) mRNA, formed by alternative splicing of exon11, were equally and robustly downregulated after hyperinsulinemia and partially recovered by serum starvation (Figure 4A). The ratio of *Insr*‐*A* and *Insr*‐*B* mRNA was not affected by hyperinsulinemia or serum starvation in cultured muscle cells (Figure 4B). Consistent with this, when we re‐processed data from one of our large human cohort,^35^ using an exon‐specific map, we observed that the abundance of exon 11 in skeletal muscle did not correlate with fasting insulin (Figure S3D). Insulin‐like growth factor 1 receptor (*Igf1r*), which has a similar structure and signaling mechanism as INSR and forms functional heterodimers with INSR,^55^ was also reduced by hyperinsulinemia at the transcriptional level (Figure 4C). Therefore, the loss of *Insr* was not compensated by *Igf1r* under these conditions nor related to differential splicing of the *Insr* gene, consistent with in vivo observations using the largest available differential exon usage analysis.^35^

Forkhead box protein O1 (FOXO1) is a known transcriptional regulator of the *Insr* gene and is also a key mediator of insulin signaling.^56^, ^57^, ^58^ In *Drosophila* and mouse myoblasts, FOXO1 activity is necessary and sufficient to increase *Insr* transcription under serum fasting and reverse this effect in the presence of insulin.^57^ We noted that genes downregulated by hyperinsulinemia belonged to the ‘FOXO signaling pathway’ (FDR = 9.95 × 10^−4^), and most of these genes were recovered by serum starvation (Figures 4D and S2B). Therefore, we sought to determine the activity of FOXO1 in our hyperinsulinemic model (Figure 4E). Insulin increased FOXO1 phosphorylation on T24, which is an AKT‐associated event known to exclude FOXO1 from the nucleus, decreasing its transcriptional activity,^59^ without altering total FOXO1 abundance (Figure 4E). T24 phosphorylation of FOXO1 decreased after starvation (Figure 4E), consistent with our observed effects on AKT phosphorylation and *Insr* transcription. Our data, therefore, support the work of other groups indicating a role for FOXO1 in *Insr* gene expression. However, knocking down *Foxo1* mRNA by 40% did not alter *Insr* mRNA level in C2C12 myoblast (*n* = 5, Figure 4F). Although we were unable to achieve a greater knockdown, this reduction reflects what might be expected from a physiological change, as our mouse model with more insulin (*Ins1* ^+/+^; *Ins2* ^−/−^ vs. *Ins1* ^+/−^; *Ins2* ^−/−^) had a ~20% decrease in *Insr* mRNA and a trend of a ~50% decrease in *Foxo1* mRNA.^13^ This result may indicate redundancy in terms of transcriptional control of *Insr*, or that the remaining 60% of *Foxo1* expression was sufficient to maintain *Insr* gene expression.

### 3.5 Hyperinsulinemia reduces INSR protein abundance but not its phosphorylation or internalization

**FIGURE 5:**
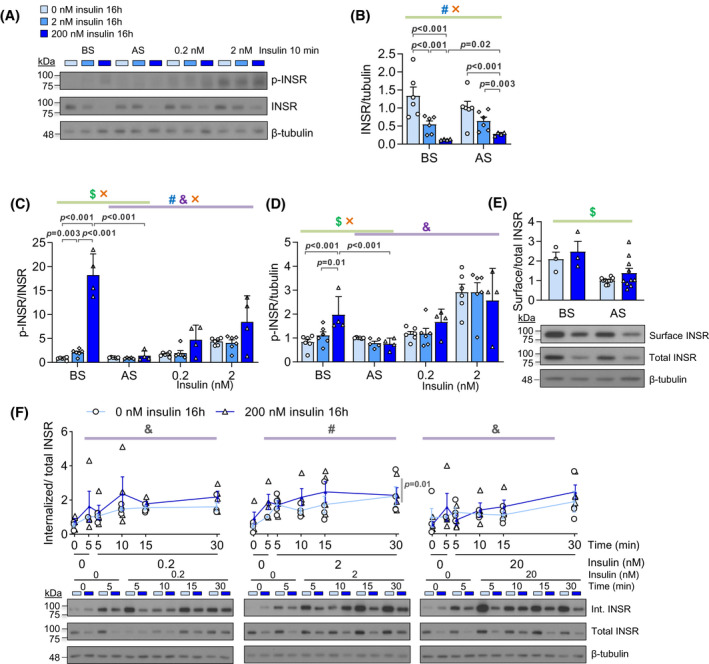
Effects of hyperinsulinemia and serum starvation on INSR abundance, phosphorylation, and internalization. (A) Representative western blot images of phospho‐INSR (Y1150/1151) and total INSR. (B) The level of total INSR protein before or after serum starvation (*n* = 4–6). The ratio of phospho‐INSR (Y1150/1151) to total INSR (C) or tubulin (D) before starvation (BS), after starvation (AS), and stimulated by 0.2 or 2 nM insulin for 10 min after serum starvation (*n* = 4–6). (E) The ratio of surface to total INSR (*n* = 3 in BS group, *n* = 10 in AS group). (F) The ratio of internalized to total INSR over 30 min under 0.2, 2 or 20 nM acute insulin stimulation (*n* = 4). (^#^effect of hyperinsulinemia, ^$^effect of starvation, ^×^interaction between two factors, Mixed Effect Model)

To further examine the direct effects of hyperinsulinemia on the proximal stages of insulin signaling, we examined INSR abundance, phosphorylation and internalization in cultured muscle cells. Consistent with the reduction in *Insr* mRNA, total INSR protein abundance was robustly decreased in both 2 and 200 nM hyperinsulinemia groups in an insulin dose‐dependent manner (Figure 5A,B). Serum starvation slightly recovered the INSR downregulation in 200 nM insulin group (Figure 5B). These results clearly demonstrated that prolonged insulin directly modulates INSR abundance in this cell system – consistent with in vivo clinical correlations.

We also examined INSR tyrosine 1150/1151 autophosphorylation, which is an early step of insulin signaling that recruits IRS and SHC, leading to PI3K‐AKT or RAS‐ERK activation.^48^ Before starvation, both hyperinsulinemia groups had increased INSR phosphorylation, suggesting that there was continuous insulin signaling during the high insulin treatments (Figure 5C,D). Serum starvation completely reversed INSR hyperphosphorylation (Figure 5C,D). While INSR phosphorylation was not significantly different after 10 min of acute insulin stimulation (Figure 5C,D), analysis of dose‐ and time‐dependent insulin signaling revealed a tendency for increased phosphorylated‐to‐total INSR ratio in insulin‐stimulated cells exposed overnight to 200 nM insulin (Figure S1C). The increased INSR phosphorylation per receptor was offset by the reduced INSR number, leading to a decreased phospho‐INSR‐to‐tubulin ratio (i.e. the overall INSR phosphorylation events per cell) (Figure S1C). These data indicate that there were no defects in INSR phosphorylation upon acute insulin stimulation in our system, but that the INSR abundance could limit the overall amount of phosphorylated INSR.

Impaired INSR endocytosis has been implicated in insulin resistance.^60^, ^61^ Many genes related to endocytosis pathways were downregulated by hyperinsulinemia (BS, 200 vs. 0 nM) and recovered by starvation (200 nM, AS vs. BS) (Figure S4A). Therefore, basal surface INSR, as well as dose‐ and time‐dependent INSR internalization were examined in our hyperinsulinemia model using a surface biotinylation assay (Figure S4B). Serum starvation slightly decreased the surface‐to‐total INSR ratio, while hyperinsulinemia had no significant effects (Figure 4E). Upon acute insulin stimulation, the internalized INSR to total INSR ratio did not have evident differences except for a small increase when stimulated by 2 nM insulin (Figure 4F). Therefore, hyperinsulinemia‐induced insulin resistance may be mediated by a reduction in total INSR that results in a proportional reduction in INSR protein at the cell surface. The fraction of INSR internalized during acute insulin signaling seemed to be recalibrated instead of drastically affected by hyperinsulinemia under these conditions. Collectively, our experiments suggest that hyperinsulinemia‐induced insulin resistance in muscle cells is mediated by a reduction in total INSR, and not primarily by affecting its activity or internalization.

### 3.6 Circulating insulin negatively correlates with INSR protein level in vivo

**FIGURE 6:**
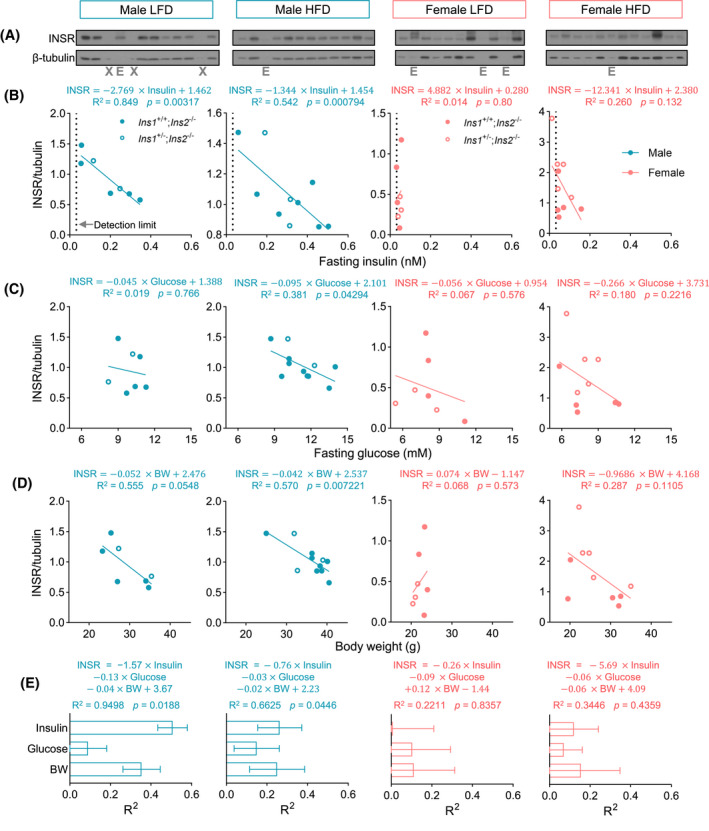
In vivo correlation between INSR abundance, fasting insulin, and glucose in skeletal muscle. (A) Western blots of INSR and β‐tubulin detected in the skeletal muscle lysates from male or female mice fed with LFD or HFD. ‘X’ indicates empty lanes, and ‘E’ indicates excluded lanes due to low protein. (B–D) Linear regression between INSR protein abundance and (B) fasting insulin, (C) fasting glucose, or (D) body weight (BW) in male or female mice fed with LFD or HFD. (E) In the multiple linear regression models using fasting insulin, glucose, and body weight as covariates and INSR as response variable, the contributions (general dominance) of fasting insulin, fasting glucose and body weight to the models were calculated by the Dominance Analysis and shown as the *R* ^2^ of each covariate that contributed to total *R* ^2^. (*n* = 7–11)

To further extend our in vitro studies, we examined the relationship between in vivo insulin concentration and muscle INSR protein abundance in mice. As in our previous studies,^13^ insulin gene dosage was manipulated to generate greater variance in circulating insulin. The mice were fed with a HFD known to induced pronounced hyperinsulinemia^13^, ^62^ or a LFD for 12 weeks. Fasting insulin and glucose were higher in male mice. INSR abundance in skeletal muscle were detected by western blot (Figure 6A). Linear regression showed that, in male mice, INSR protein abundance negatively correlated with fasting insulin and body weight in LFD and HFD group (Figure 6B,D) but only negatively correlated with fasting glucose in the HFD group with weaker correlation (Figure 6C). On the other hand, in female mice, fasting insulin levels were near or at the lower detection limit, reducing measurement dynamic range, and had a lack of correlation with INSR (Figure 6B). Fasting glucose levels and body weight also did not significantly correlate with INSR (Figure 6B). Multiple linear regression showed that fasting insulin, fasting glucose and body weight can explain around 95% (*R* ^2^ = 0.9498) or 66% (*R* ^2^ = 0.6625) of the variance of INSR abundance in the male LFD or HFD group, respectively. However, those predictors poorly modeled INSR in female mice (Figure 6E). In the male LFD group, dominance analysis indicated that fasting insulin had the largest contribution to the regression model of INSR, followed by body weight and fasting glucose, which had a low contribution (Figure 6E). In the male HFD group, both fasting insulin and body weight had equally moderate contributions, but higher contribution than fasting glucose, to the regression model of INSR (Figure 6E). Together, these data support the concept that insulin is a strong negative regulator of INSR independent from glucose in skeletal muscle. This is consistent with our in vitro hyperinsulinemia model and our previous in vivo data demonstrating improved insulin sensitivity over time in mice with genetically reduced insulin production.^12^ Body weight, which can be driven by insulin,^63^ is another negative predictor of INSR. These data also suggest an interaction between insulin, glucose and INSR that is dependent on the conditions of the HFD. The source of the aforementioned sex differences may again reflect insulin dose‐dependent effects and requires further investigation.

### 3.7 Novel transcription factors regulate *Insr* expression and transcriptomic remodeling by insulin

**FIGURE 7:**
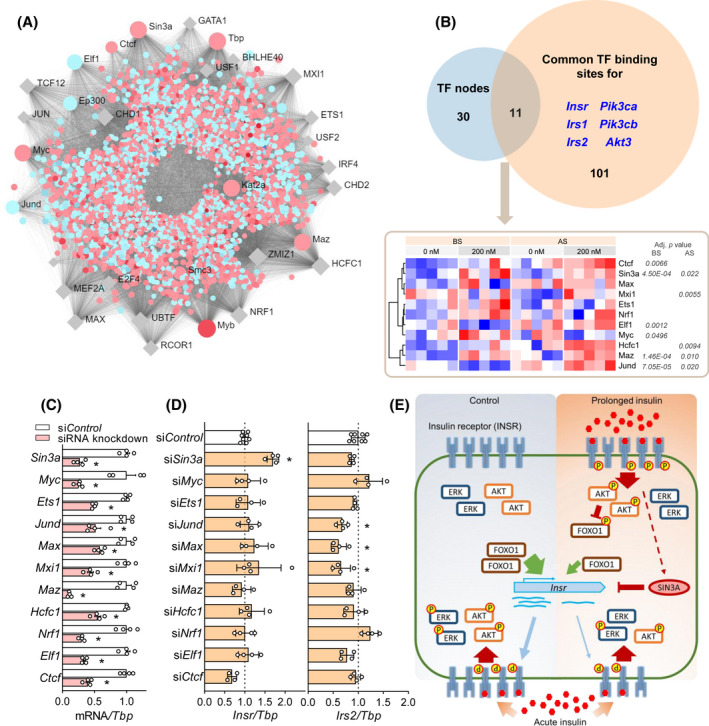
Identification of upstream transcription factors mediating the overall transcriptomic changes and insulin receptor expression. (A) Transcription factor (TF)‐gene network predicting upstream transcriptional regulators of differentially expressed genes by hyperinsulinemia (BS, 200 vs. 0 nM). Names of the top 30 TFs are labeled. Genes that are up or down‐regulated are shown in red or blue dots, respectively. TFs that are not differentially expressed are in grey rhombus. (B) Common TFs between the top 30 TF nodes from (A) and the TF binding sites for several key genes in insulin signaling pathways that are downregulated by hyperinsulinemia. Adjusted *p* values of differentially expressed TFs (200 vs. 0 nM, both BS and AS) are labeled next to the heatmap. (C) mRNA levels of the common TFs in (B) after siRNA knockdown. (D) The effects of each siRNA knockdown on *Insr* and *Irs2* mRNA levels. (*n* = 4. *p < .05, 1‐ANOVA followed by Dunnett multiple comparison test against si*Control*.) (E) Graphic summary of our current model. Hyperinsulinemia induced sustained phosphorylation of INSR and AKT, which resulted in the inhibition of FOXO1 leading to reduced *Insr* transcription. Downregulated INSR and post‐receptor components resulted in reduced insulin signaling upon acute insulin stimulation. SIN3A, which was upregulated by hyperinsulinemia, represses *Insr* transcription

We identified upstream transcriptional regulatory proteins by examining transcription binding sites in the differentially expressed genes using ENCODE ChIP‐seq data (Figure 7A). The top 30 transcription factors with high degrees of connections to altered genes (Figure 7A), were compared to common transcription factor binding sites of several differentially expressed genes that encode key proteins in insulin signaling (Figure 7B). The resulting 11 transcription factors all have binding sites near the transcription start sites of the selected insulin signaling genes based on the ENCODE ChIP‐seq data and were deemed candidates to affect the transcriptional changes in *Insr* and the other selected insulin signaling genes during hyperinsulinemia (Figure 7B). Among them, *Sin3a*, *Myc* and *Ets1* were upregulated by in vitro hyperinsulinemia (Figure 7B). To investigate the role of these transcription factors on *Insr* expression, we conducted siRNA knockdown for each transcription factor, with knockdown efficiencies varying between 45% and 85% (Figure 7C). *Sin3a* knockdown (~70%) resulted in a significant increase in *Insr* mRNA, indicating that this transcription factor has a repressive effect (Figure 7D). In addition, we assessed the expression of *Irs2*, which had a larger fold change than *Insr* and was one of the most significantly altered genes upon hyperinsulinemia and starvation (Figure S2A). Knockdown of *Jund*, *Max* and *Mxi1* downregulated *Irs2*, which suggested that these transcription factors are involved in *Irs2* transcription and may contribute to insulin resistance (Figure 7D). In conclusion, we identified transcription factors for *Insr* and *Irs2* genes among the predicted upstream transcriptional regulators.

## 4 DISCUSSION

The goal of this study was to explore the mechanisms of hyperinsulinemia‐induced insulin resistance in skeletal muscle cells with a focus on transcriptomic changes. We demonstrated that prolonged physiological and supraphysiological hyperinsulinemia induced a reduction of AKT and ERK signaling and insulin‐stimulated glucose uptake (Figure 7E). Remarkably, while serum starvation partially reversed the effects of overnight hyperinsulinemia, much of the impaired acute insulin signaling and transcriptomic remodeling was sustained after 6 h of insulin withdrawal and serum starvation, suggesting that stable molecular changes underlie these differences. The effects of prolonged hyperinsulinemia were insulin dose‐dependent from the physiological to the supraphysiological range. We demonstrated that the impaired insulin response in our system can be partially accounted for by INSR downregulation at the transcription level and also used transcriptomic profiling to discover new factors that regulate insulin signaling in our system including SIN3A, JUND, MAX and MXI1. These experiments showcased many genes that were reprogramed by hyperinsulinemia and insulin removal.

Our in vitro cell culture model provided a robust and controlled system for examining the direct effects of excess insulin, and insulin withdrawal, on multiple components of insulin signaling in muscle cells. Our results are consistent with other in vitro cell culture systems designed to examine the effects of hyperinsulinemia. For example, reduced AKT and ERK signaling and INSR abundance were also reported in hyperinsulinemia‐treated β‐cells (INS1E cell line and rat islets) and enteroendocrine L cells.^64^, ^65^ Nevertheless, the mechanisms of sustained alterations in AKT and ERK phosphorylation were not fully understood. In our in vitro model, AKT and ERK phosphorylation was suppressed at all time points during 30‐min acute insulin stimulation, suggesting that the insulin resistance we observed was impaired responsiveness, consistent with signaling deficiencies at both the receptor level and in post‐receptor components.^66^ Indeed, multiple components of insulin signaling were reduced at the transcriptional level revealed by our transcriptomics analysis. Our observations also verified the distinct responses to hyperinsulinemia on the bifurcate insulin signaling pathways. Chronic 200 nM insulin treatment preferentially increased basal AKT phosphorylation, as a sign of sustained activation, but did not increase the basal ERK phosphorylation, possibly due to desensitization, as reported in neurons.^67^ Diet‐ and hyperinsulinemia‐induced insulin resistance is generally considered to be related specifically to AKT phosphorylation. Chemical inhibition of the AKT pathway using the non‐selective PI3K inhibitor LY‐294002, but not ERK pathway inhibition, has been reported to protect from insulin resistance both in vitro and in vivo.^67^, ^68^ Further work is required to understand the interplay between INSR expression and both major branches of downstream signaling. Beyond signal transduction, we also demonstrated that hyperinsulinemia could directly reduce insulin‐stimulated glucose uptake, a function of insulin. The physiological consequences of altered insulin signaling on glucose uptake and storage may be best studied in vivo, rather than in this cell line.

A major observation of our work is that *Insr* mRNA was directly reduced by hyperinsulinemia in cultured cells, consistent with reports from other cell culture systems,^52^, ^53^ and also is consistently negatively correlated with fasting insulin in both mouse and human skeletal muscle in vivo. Indeed, T2D patients were found to have lower *INSR* mRNA expression in skeletal muscle biopsies.^69^ Notably, hyperglycemia can increase *Insr* expression in lymphocyte and cancer cell lines,^70^, ^71^ while high glucose inhibits β‐cell *Insr* expression through autocrine insulin action and INSR‐FOXO1 signaling.^70^, ^71^ Interestingly, glucose only induces insulin resistance in the presence of insulin in cultured hepatocytes, adipocytes and skeletal muscle cells.^72^, ^73^, ^74^ Therefore, reduced *Insr* expression by hyperinsulinemia may be a key, independent factor of INSR downregulation and insulin resistance.

Intermittent fasting, time‐restricted feeding, caloric restriction, and carbohydrate restriction positively modify risk factors in diabetes, including reducing hyperinsulinemia, increasing insulin sensitivity, improving β‐cell responsiveness, and lowering the levels of circulating glucose.^75^, ^76^, ^77^ Several human trials suggest that fasting regimes can be more effective for reducing insulin and increasing insulin sensitivity than they are for reducing glucose.^78^, ^79^ By mimicking the low‐insulin state, the serum starvation phase of our studies revealed some possible molecular mechanisms of the beneficial effects of fasting on muscle cells, including the restoration of protein phosphorylation in insulin signaling pathways and partial recovery of *Insr* transcription, INSR protein and overall transcriptomic changes. These data hint that some deleterious effects of hyperinsulinemia are reversible but may require a long enough period of reduced insulin exposure to ‘reset’.

Pathway analysis of the transcriptomics correctly revealed broad effects of hyperinsulinemia and serum starvation on insulin signaling and FOXO signaling pathways and highlighted potential upstream transcription factors. This further supports utility of robust RNA pathway analysis alone to correctly identify key protein regulators in muscle tissue.^80^ Besides FOXO1, other transcription factors such as SP1, HMGA1, C/EBPβ and NUCKS have been reported to regulate *Insr* expression.^81^, ^82^, ^83^ We identified at least one novel transcriptional repressor of the *Insr* gene, SIN3A, which was upregulated by hyperinsulinemia. SIN3A interacts with histone deacetylases, typically HDAC1/2, to inhibit transcription and interacts with other transcription factors that were identified in our informatics analyses.^84^ For example, SIN3A and MYC inhibit each other and form a negative feedback loop.^85^ MAX dimerizes with either MYC or MXI1 (MAD family protein) in a competing manner to activate or repress target genes,^86^ and MXI1 recruits SIN3A for gene inhibition.^86^ Interestingly, the knockdown level we achieved for MYC, MAX, MXI1 did not have significant effects on the transcription of *Insr* in muscle. One possibility is that the roles of these transcription factors on *Insr* are indirect and rely on the action of SIN3A, while another possibility is that SIN3A acts through alternative pathways. A recent study identified SIN3A as a FOXO1 corepressor of the glucokinase gene in the liver,^87^ and this may represent a possible node of interaction in our system. Interestingly, *Irs2* is a known target of FOXO1,^88^, ^89^ and it was not altered in our *Sin3a* knockdown cells. Therefore, the regulation of *Insr* mediated by SIN3A and/or FOXO1 seems to be gene‐specific and requires more investigation.

Despite its inherent reductionism, our in vitro model identified plausible molecular features underpinning the descriptive relationship between hyperinsulinemia in the development of insulin resistance and T2D. The transcriptomic responses in our muscle cell model were reflective of the correlation analysis of human skeletal muscle, indicating that it represents an informative resource to interrogate hyperinsulinemia‐induced insulin resistance. We demonstrated that in vitro hyperinsulinemia and serum ‘fasting’ have profound effects on AKT and ERK signaling, INSR abundance and localization, and transcriptional activities. Beyond altered transcription of *Insr* gene, proteosomal and lysosomal degradation of INSR are additional mechanisms for reduced protein expression. A recent study demonstrated that the E3 ligase, MARCH1, can specifically ubiquitinate surface INSR, leading to their internalization and proteosomal degradation.^90^ In a neuronal cell line, 24‐h insulin exposure induced blunted AKT signalling and lysosomal degradation of INSR but did not decrease *INSR* mRNA.^91^ Lysosomal, but not proteosomal, inhibitor prevented the INSR downregulation and partially rescued AKT signalling. In addition to the downregulation of INSR protein abundance, we found a subtle increase in INSR internalization, and future studies will be required to determine the importance and mechanisms of this phenomenon. Future additional characterization of the effect of hyperinsulinemism on INSR trafficking, degradation, and detailed post‐receptor alterations on protein level will provide a greater understanding of the role of hyperinsulinemia in promoting obesity^9^ and diabetes.

## DISCLOSURES

The authors declare that they have no conflicts of interest with the contents of this article.

## AUTHOR CONTRIBUTIONS

Haoning Howard Cen designed the study, performed all experiments except the high‐fat feeding, analyzed data, and wrote the manuscript. Bahira Hussein performed the glucose uptake experiments, analyzed related data, and edited the manuscript. José Diego Botezelli performed in vivo high‐fat feeding and analyzed the related data. Su Wang performed the statistical analysis and edited the manuscript. Jiashuo Aaron Zhang performed the linear regression analysis and dominance analysis and edited the manuscript. Nilou Noursadeghi performed siRNA knockdown experiments. Niels Jessen contributed to the RNAseq data and edited the manuscript. Brian Rodrigues provided the key cell line in the study and guidance on the glucose uptake experiment. James A. Timmons contributed to the design of the transcriptomic analysis and edited the manuscript. James D. Johnson designed the study, supervised the research, and edited the manuscript. James D. Johnson is the ultimate guarantor of this work.

## Supporting information

Supplementary Material

Dataset S1

## Data Availability

The new RNA‐seq data from the cell experiments are available under GEO accession GSE147422. All remaining data are contained within the article and can be shared upon request or at GEO (GSE154846). Code for all the analyses is available at GitHub deposit https://github.com/hcen/Hyperinsulinemia_muscle_INSR.
