# Supplementary material for: Human and mouse muscle transcriptomic analyses identify insulin receptor mRNA downregulation in hyperinsulinemia‐associated insulin resistance
Source: FASEB J. 2021 Dec 18;36(1):e22088. doi: 10.1096/fj.202100497RR (PMC9255858; doi:10.1096/fj.202100497RR)
Supplement: Supplementary file 1 — Supplementary Material [file FSB2-36-0-s001.pdf]

Supplementary figure legends

Figure S1. Insulin dose- and time-depend acute signaling in the hyperinsulinemia-induced insulin resistance model. Myotubes cultured in control (0 nM insulin) or hyperinsulinemic (200 nM insulin) medium were stimulated with acute 0.2, 2 or 20 nM insulin for 1, 5, 10, 15 or 30 min after serum starvation. **(A)** phospho-AKT (T308, S473), **(B)** phospho-ERK1/2, and **(C)** INSR phosphorylation were measured. (n=4; $^{\#}$ effect of hyperinsulinemia, $^{\&}$ effect of acute insulin, × interaction between two factors, Mixed Effect Model.)

Figure S2. RNA-seq analysis of hyperinsulinemia and serum starvation highlighting glucose metabolism and FOXO signaling pathways. (A) Top 50 most significantly altered genes with lowest p value. **(B)** Selected KEGG pathways enriched from genes downregulated by hyperinsulinemia before starvation (BS, 0 vs 200 nM insulin), which includes "FOXO signaling pathway". **(C)** The log2 fold change of the common of DE genes before starvation (BS, 200 vs 0 nM) and starvation after hyperinsulinemia (ST, 200 nM, AS vs BS). **(D)** Upregulated genes (BS, 200 vs 0 nM) enriched under Reactome pathway "Glucose metabolism". **(E)** Heatmap showing relative expression levels of genes under "Glucose metabolism" and "Glucolysis" Reactome pathways. Unmarked genes were differentially expressed by hyperinsulinemia only before starvation (BS, 200 vs 0 nM) but not after starvation. Genes marked by * were differentially expressed both before and after starvation (BS & AS, 200 vs 0 nM). Genes marked by # were only altered by starvation (200 nM, AS vs BS) but not by hyperinsulinemia. **(F)** The log2 fold change of the common of DE genes before (BS, 200 vs 0 nM) and after starvation (AS, 200 vs 0 nM).

Figure S3. Mouse and human skeletal muscle data sets. (A) Common DE genes in mouse IRMOE model and our hyperinsulinemia model (BS, 200 vs 0 nM). **(B)** Common DE genes in mouse hyperinsulinemic clamp study and our hyperinsulinemia model (BS, 200 vs 0 nM). **(C)** Fasting insulin levels of subjects in the 3 human data sets. **(D)** The correlations between the *INSR* exons and fasting insulin in human skeletal muscle.

Figure S4. Endocytosis-related differentially expressed genes and surface biotinylation assay. (A) Differentially expressed genes related to endocytosis pathways. **(B)** Scheme of surface biotinylation assay to measure surface or internalized INSR. Surface or internalized INSR in protein lysates were detected by western blots.

Table S1. Differentially expressed genes by *in vitro* hyperinsulinemia and starvation

Table S2. Reactome pathway enrichment from differentially expressed genes

Table S3. KEGG pathway enrichment from differentially expressed genes

Table S4. Comparison of differentially expressed genes in our *in vitro* model, IRMOE and clamp studies

Table S5. Comparison of insulin-correlated genes in SMP and FUSION human skeletal muscle and differentially expressed genes in our *in vitro* model

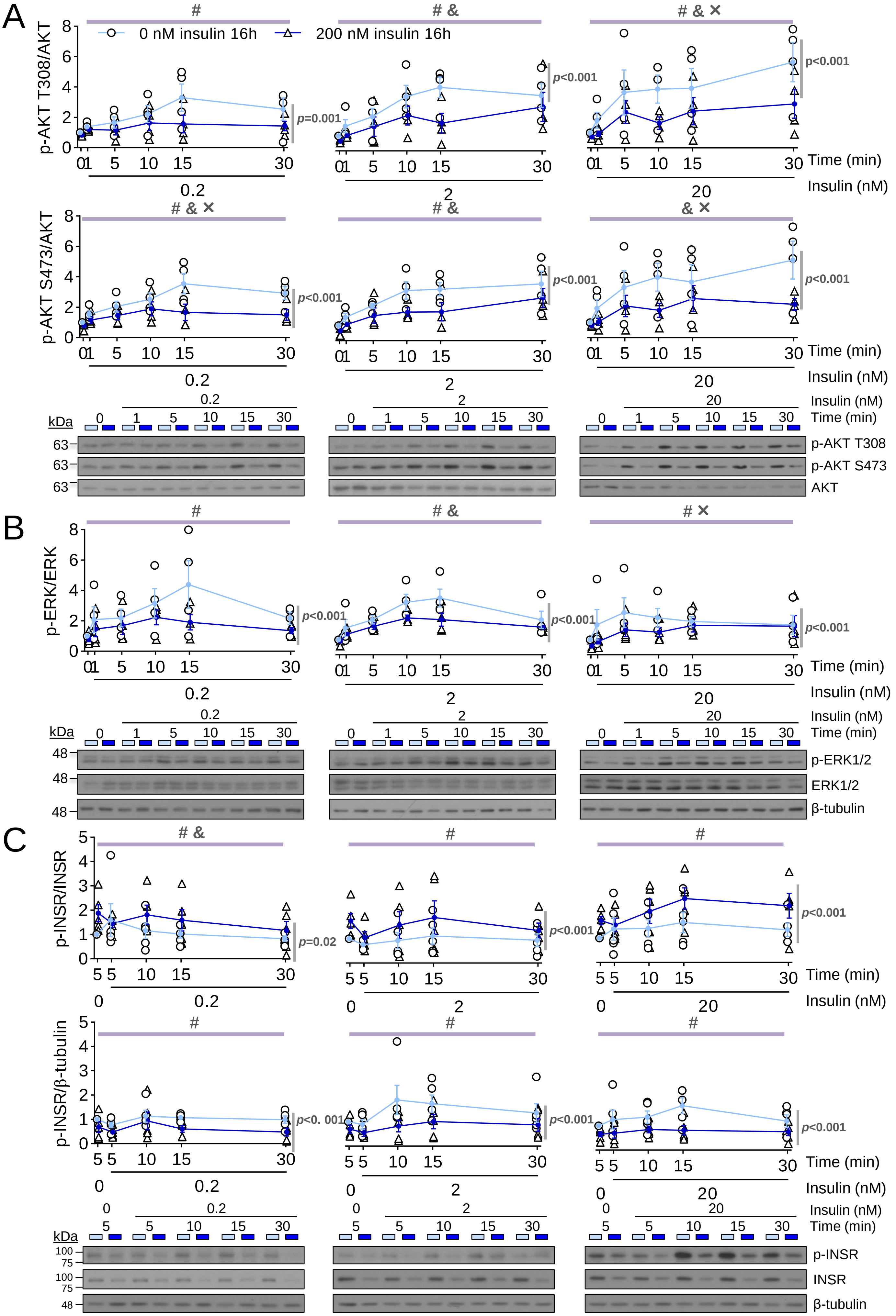

Figure S1. Insulin dose- and time-depend acute signaling in the hyperinsulinemia-induced insulin resistance model.

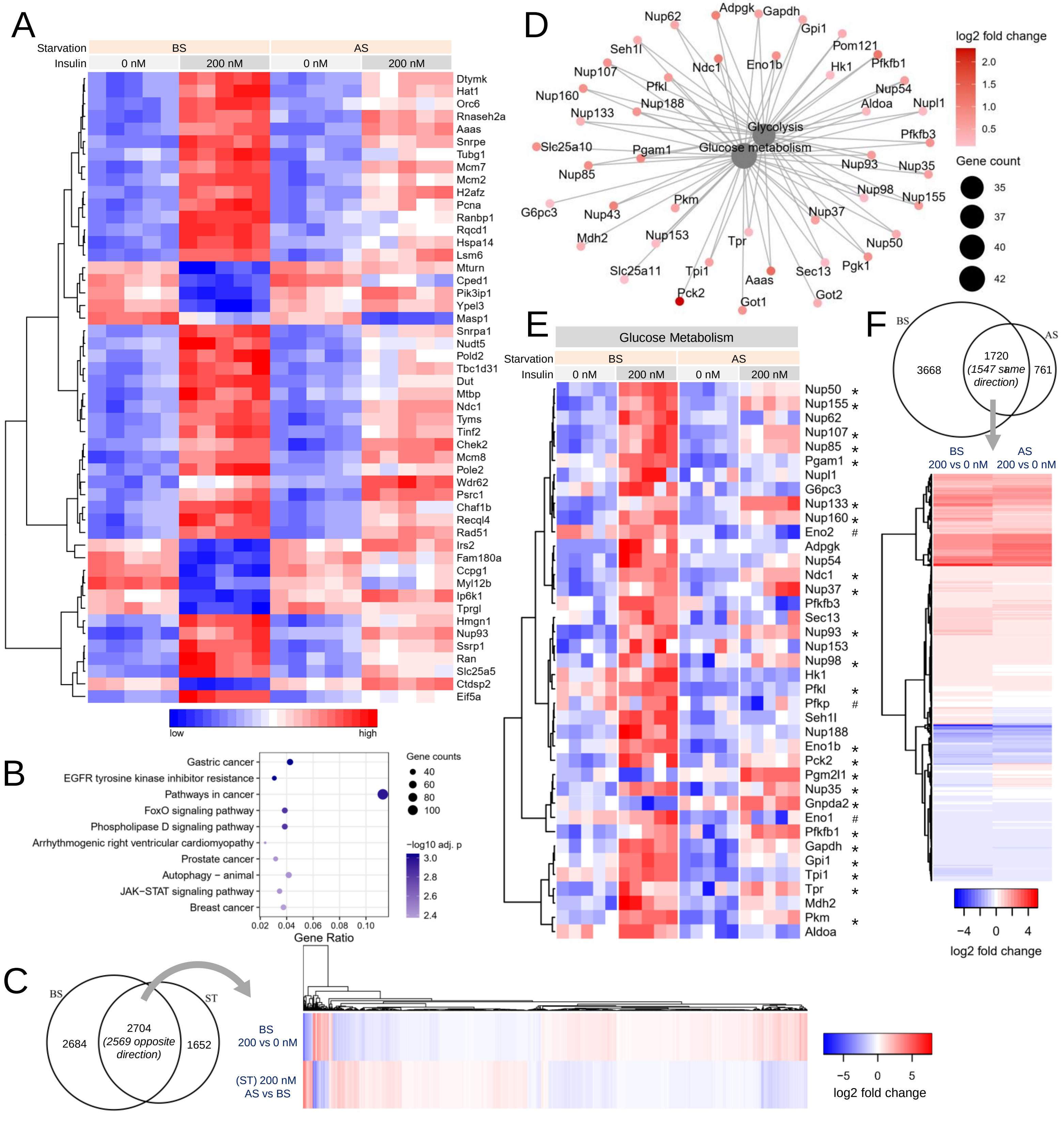

Figure S2. RNA-seq analysis of hyperinsulinemia and serum starvation highlighting glucose metabolism and FOXO signaling pathways.

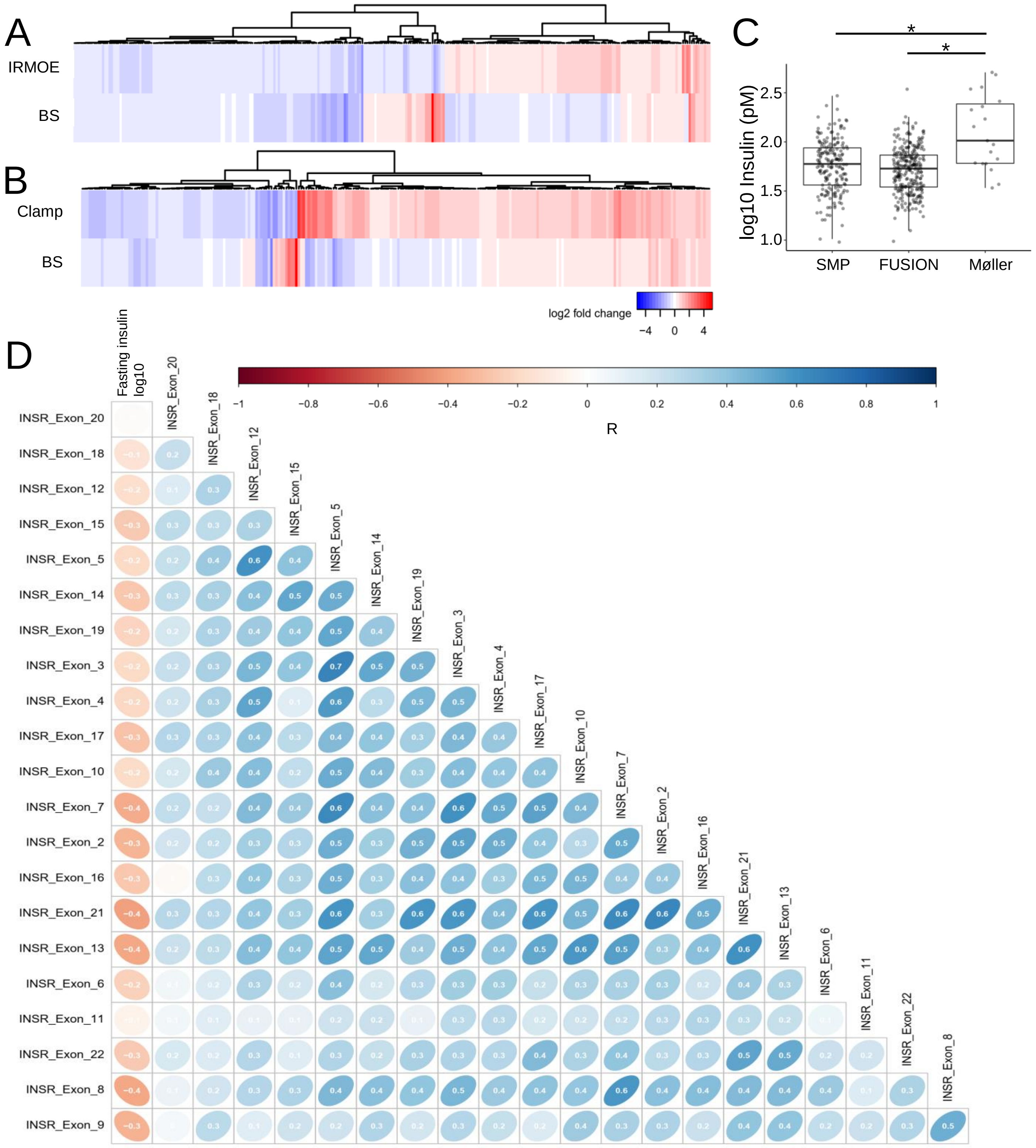

Figure S3. Mouse and human skeletal muscle data sets.

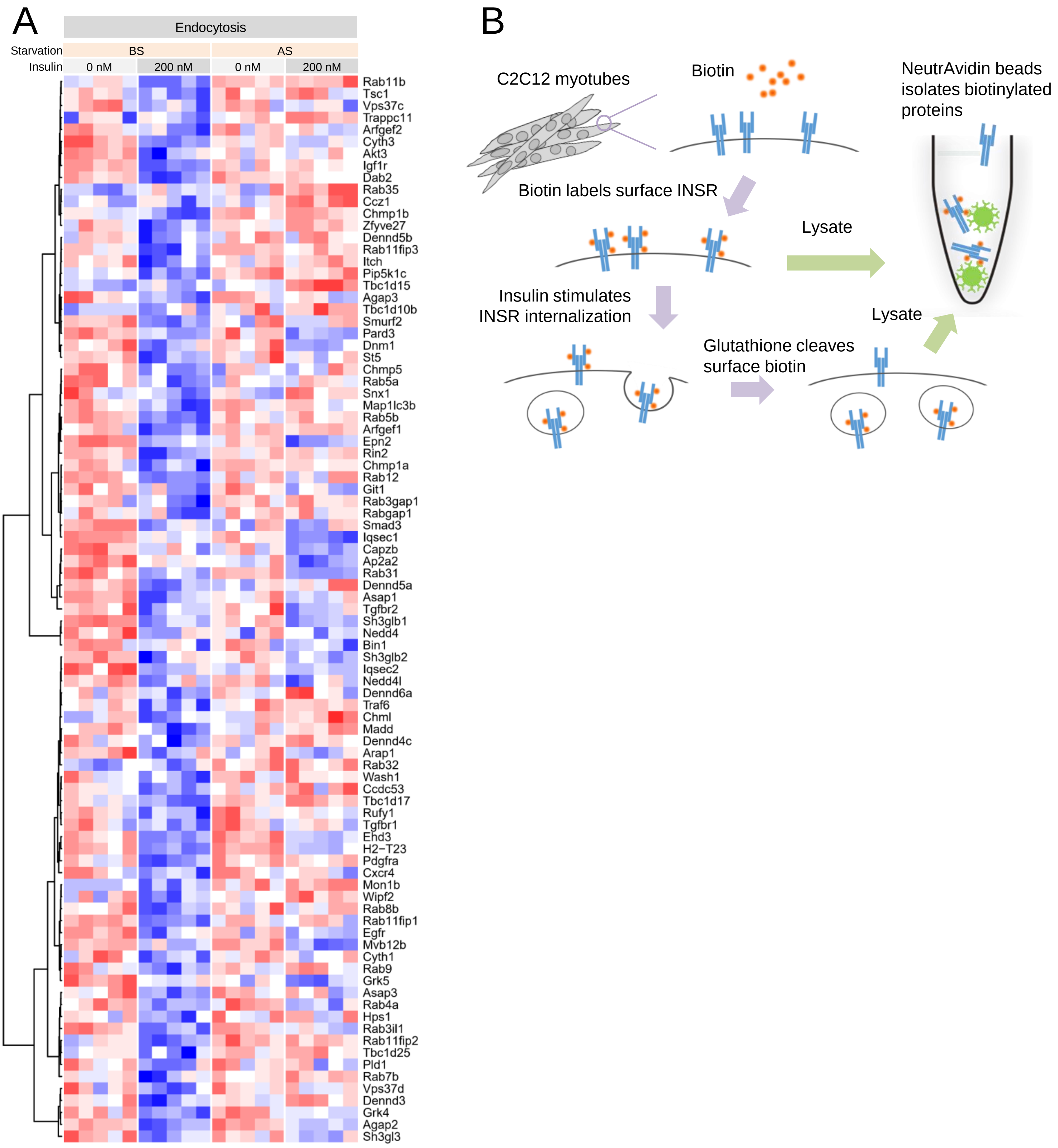

Figure S4. Endocytosis related differentially expressed genes and surface biotinylation assay.
